# *Anopheles stephensi* Heme Peroxidase HPX15 Suppresses Midgut Immunity to Support *Plasmodium* Development

**DOI:** 10.3389/fimmu.2017.00249

**Published:** 2017-03-14

**Authors:** Mithilesh Kajla, Parik Kakani, Tania Pal Choudhury, Vikas Kumar, Kuldeep Gupta, Rini Dhawan, Lalita Gupta, Sanjeev Kumar

**Affiliations:** ^1^Molecular Parasitology and Vector Biology Laboratory, Department of Biological Sciences, Birla Institute of Technology and Science (BITS), Pilani, India; ^2^Department of Zoology, Ch. Bansi Lal University, Bhiwani, India; ^3^Department of Biotechnology, Ch. Bansi Lal University, Bhiwani, India

**Keywords:** *Anopheles stephensi*, heme peroxidase, HPX15, mucin barrier, midgut, *Plasmodium*, innate immunity, vectorial capacity

## Abstract

The heme peroxidase HPX15 is an evolutionary conserved anopheline lineage-specific gene. Previously, we found that this gene is present in the genome of 19 worldwide distributed different species of *Anopheles* mosquito and its orthologs are absent in other mosquitoes, insects, or human. In addition, 65–99% amino acid identity among these 19 orthologs permitted us to hypothesize that the functional aspects of this gene might be also conserved in different anophelines. In this study, we found that *Anopheles stephensi* AsHPX15 gene is mainly expressed in the midgut and highly induced after uninfected or *Plasmodium berghei*-infected blood feeding. RNA interference-mediated silencing of midgut AsHPX15 gene drastically reduced the number of developing *P. berghei* oocysts. An antiplasmodial gene nitric oxide synthase was induced 13-fold in silenced midguts when compared to the unsilenced controls. Interestingly, the induction of antiplasmodial immunity in AsHPX15-silenced midguts is in absolute agreement with *Anopheles gambiae*. In *A. gambiae*, AgHPX15 catalyzes the formation of a dityrosine network at luminal side of the midgut that suppresses the activation of mosquito immunity against the bolus bacteria. Thus, a low-immunity zone created by this mechanism indirectly supports *Plasmodium* development inside the midgut lumen. These indistinguishable functional behaviors and conserved homology indicates that HPX15 might be a potent target to manipulate the antiplasmodial immunity of the anopheline midgut, and it will open new frontiers in the field of malaria control.

## Introduction

*Plasmodium* completes its sexual life cycle inside the mosquito where various stages of parasite develop in different body compartments of the insect host. *Plasmodium* gametocytes start the insect cycle in mosquito midgut and produce male and female gametes. Subsequently, the fertilization leads to the formation of zygote that after 16–20 h of ingestion is transformed into the motile ookinete (1). Approximately after 24 h of ingestion, the ookinetes traverse the midgut epithelium and are then transformed into the oocysts. Furthermore, in the next 10 days, several rounds of mitosis produce thousands of sporozoites. Mature sporozoites are released into the mosquito hemocoel and reach the salivary glands. Inoculation of these sporozoites into a vertebrate host continues the asexual cycle of *Plasmodium* development.

The success of *Plasmodium* sexual cycle depends on its interactions with the host immunity and internal environment. Thus, to develop transmission blocking strategies, the molecular understanding of these interactions is greatly demanded. So far, a large number of mosquito immune molecules have been identified that regulate mouse malaria parasite *Plasmodium berghei* development; however, they are ineffective against human malaria parasite *Plasmodium falciparum* (2–4). Thus, the discovery of those mosquito molecules, which can regulate the development of human malaria, requires sincere efforts. Recent studies identified that, in African mosquito *Anopheles gambiae*, the heme peroxidases play an important role in the regulation of *Plasmodium* development. One of the *A. gambiae* heme peroxidase, AgHPX2, along with AgNOX5 (NADPH Oxidase 5) catalyzes the nitration of epithelial cells that reduces the development of *P. berghei* ookinetes (5). In addition, another *A. gambiae* heme peroxidase AgHPX15 cross-links the mucins barrier on the luminal side of the midgut epithelium and that, in turn, blocks the recognition of *Plasmodium* by the mosquito innate immunity (6, 7). This mechanism, in fact, is an innate process that protects naturally acquired midgut microbes against the mosquito immunity and *Plasmodium* takes an advantage of this event (6–8). Silencing of AgHPX15 gene suppressed *P. berghei* as well as *P. falciparum* oocysts development due to the reduced integrity of the mucin barrier and activation of antiplasmodial midgut immunity (6).

Our previous studies identified AgHPX15 ortholog in major Indian malaria vector *Anopheles stephensi*, and we termed it as AsHPX15 (9, 10). In addition, putative orthologs of AsHPX15 gene were also found in the genome of 17 other anophelines with no orthology in other insects, mosquitoes (*Aedes* and *Culex*), or human. These findings revealed that HPX15 is an anopheline lineage-specific unique gene. Interestingly, these 19 HPX15 orthologs are also highly conserved and reveal 65–99% amino acid identity among them (10, 11). Based on these facts, we hypothesized that HPX15 might be functionally conserved in anophelines and can be considered as a general target to block the insect cycle of *Plasmodium* development. However, this hypothesis demands further investigations to establish the aforesaid regulatory role of HPX15 gene, in terms of *Plasmodium* development, in other anophelines. Thus, in the present study, we used the gene-silencing approach to determine the antiplasmodial role of AsHPX15 gene in the Indian malaria vector *A. stephensi*.

## Materials and Methods

### Rearing of Mosquitoes

Mosquito colony was maintained in insectory at 28°C, 80% relative humidity, and 12 h light–dark cycle as described before (12). Larvae were fed on a 1:1 mixture of dog food (PetLover’s crunch milk biscuit, India) and fish food (Gold Tokyo, India) as before (11, 12). Adult mosquitoes were fed on 10% sugar solution *ad libitum*. For colony propagation, 3–4 days old, starved females were fed on anesthetized mice. The eggs laid by these blood-fed (BF) females were collected in moist condition, and the hatched larvae were floated in water to continue the cycle.

### Malaria Parasite *P. berghei* Maintenance

The transgenic *P. berghei* ANKA strain that expresses GFP (PbGFP) in all developmental stages (13) was a gift from Dr. Agam Prasad Singh, National Institute of Immunology, New Delhi, India. The *Plasmodium* strain was maintained in Swiss albino mice following the standard protocols as before (6, 14, 15). The parasitemia of the infected mice was determined from Giemsa-stained blood smears as mentioned before (14, 15). For blood stage passages, 100–150 μl of blood from an infected mouse (containing ~10–15% parasitemia) was injected intraperitoneal into the healthy mice. Parasitemia and potential infectivity of the *Plasmodium* to the mosquitoes was determined by exflagellation assays as before (16). In brief, 2 μl blood from the tail of the infected mouse was mixed with 20 μl of exflagellation buffer that was prepared by mixing equal parts of solution A (10mM Tris-Cl, 150mM NaCl, and 10mM glucose, pH 8) and heat-inactivated fetal bovine serum. In all the experiments, mice containing ~5–7% parasitemia and 2–3 exflagellations per field under 40× objective were used to infect the mosquitoes.

### *P. berghei* Infection in Mosquito

The 4- to 5-day-old and overnight-starved 200 female mosquitoes were allowed to feed on an anesthetized Swiss albino mouse infected with the GFP expressing *P. berghei*. The mosquitoes fed on an uninfected mouse served as control. The unfed mosquitoes were removed and only fully engorged females were maintained at 21°C and 80% humidity, the permissive conditions for *P. berghei* development as discussed before (6, 11, 15, 17). Midguts samples were collected from 20 mosquitoes at different time points (3, 6, 12, 18, and 24 h) as mentioned below.

### dsRNA Synthesis

A 218-bp fragment of the lacZ gene was amplified using the following primers (5′ to 3′) Fw-GAGTCAGTGAGCGAGGAAGC and Rev-TATCCGCTCACAATTCCACA and cloned into the pCRII-TOPO vector as before (18). In parallel, a 428-bp cDNA fragment of AsHPX15 gene that reported in our previous publication (10) was also cloned in the same vector. These recombinant plasmids were used individually as a template for *in vitro* transcription. The recombinant plasmid already had a T7 promoter site at M13F primer end, thus, a T7 promoter site was added to the other end of the fragment through amplifying M13R primer. The sequences (5′-3′) for these primers are following: M13F-GTAAAACGACGGCCAGT and T7-M13R-CTCGAGTAATACGACTCACTATAGGGCAGGAAACAGCTATGAC. PCR amplification using M13F and M13R primers was started with 94°C for 5 min followed by 40 cycles at 94°C for 30 s, 55°C for 30 s, 72°C for 30 s, and final extension was carried at 72°C for 10 min. Amplicons were purified with the QIA quick PCR purification kit (Qiagen, Valencia, CA, USA). PCR-purified amplicons tailed with T7 promoter sequences were used to synthesize dsRNAs with the MEGAscript kit (Cat No. AM1626, Ambion, Austin, TX, USA) following the manufacturer’s instructions. dsRNA was further purified using a Microcon YM-100 filter (Millipore) and finally concentrated to 3 μg/μl in DNase and RNase free water.

### The Effect of dsRNA-Mediated HPX15 Gene Silencing on *Plasmodium* Development

The 1- to 2-day-old female mosquitoes were injected with 69 nl of 3 μg/μl dsAsHPX15 RNA (finally 207 ng/mosquito). Control mosquitoes were injected with dsLacZ RNA in the same manner. The gene silencing efficiency of the method was analyzed in sugar fed (SF), blood fed (BF), and *P. berghei*-infected midguts through qPCR against the respective controls. In these samples, on an average, we could achieve 98% silencing. To evaluate the effect of gene silencing on *Plasmodium* development, 4 days post dsRNA injected females were fed on transgenic GFP-*P. berghei*-infected mice, and the number of oocysts per midgut was determined after 7 days post infection. Furthermore, the midguts were dissected in Ashburner’s PBS (3mM sodium chloride, 7mM disodium hydrogen phosphate, and 3mM sodium dihydrogen phosphate, pH 7.2), fixed for 15 min with 4% paraformaldehyde, washed thrice in PBS, and mounted on glass slides in Vectashield mounting medium. The numbers of green fluorescent oocysts were counted in each midgut under a fluorescent microscope (Olympus).

### Sample Collection

Midguts from 20 uninfected or *P. berghei*-infected blood fed mosquitoes were dissected at different time points (3, 6, 12, 18, and 24 h) after the blood feeding. The dissected midguts or carcasses (rest of the body except midgut) were kept in RNAlater solution (Qiagen) and stored at −80°C. The midguts from randomly collected 20 SF mosquitoes were also dissected in similar way.

### RNA Isolation, cDNA Synthesis, and qPCR

Total RNA from the midgut samples was isolated using RNeasy Mini Kit (Qiagen Cat no. 74104) following manufacturer’s instructions. First-strand cDNA was synthesized from total RNA using QuantiTect Reverse Transcription kit (Qiagen Cat no. 205311). Expression profile of different genes was carried through semiquantitative real-time PCR using SYBR Green supermix in a CFX Connect™ real-time PCR detection system (Bio-Rad) where ribosomal protein subunit S7 mRNA was used as internal loading control for normalization as described before (11, 19). The PCR primer pairs used for different genes are mentioned in Table 1. The PCR started with an initial denaturation at 95°C for 3 min and followed by 35 additional cycles at 94°C for 10 s, 57°C for 30 s, and 72°C for 50 s. Fluorescence for the PCR products was read at 72°C after each cycle. A final extension at 72°C for 10 min was completed before deriving a melting curve. Each experiment was performed in three independent biological replicates. Relative mRNA levels were calculated using ^ΔΔ^C_t_ method by dividing the technical replicates in the control or test groups by the mean of the control group. This adjusted the control groups to a value of 1.0 as discussed before (6, 20–22).

**Table 1:** **List of primers used for real-time PCR**.

S. no.	Primer sets	Primer sequence (5′–3′)	PCR product (bp) from cDNA template	Purpose	Reference
1	AsHPX15 Fw2	GAGAAGCTTCGCACGAGATTA	329	Real-time PCR	Kajla et al. (10)
	AsHPX15 Rev2	GAATGTCGATTGCTTTCAGGTC			
2	Suppressor of cytokine signaling (SOCS) Fw	CGTCGTACGTCGTATTGCTC	456	Real-time PCR	Dhawan et al. (15)
	SOCS Rev	CGGAAGTACAATCGGTCGTT			
3	Nitric oxide synthase (NOS) Fw	ACATCAAGACGGAAATGGTTG	250	Real-time PCR	Luckhart et al. (23)
	NOS Rev	ACAGACGTAGATGTGGGCCTT			
4	Thioester-containing protein 1 (TEP1) Fw	GCTATCAAAATCAGATGCGCTATC	325	Real-time PCR	Present study
	TEP1 Rev	ATCACAACCGCATGCTTCA			
5	S7 Fw	GGTGTTCGGTTCCAAGGTGA	487	PCR internal loading controls	Vijay et al. (24)
	S7 Rev	GGTGGTCTGCTGGTTCTTATCC			

### Statistical Analysis of the Data

Statistical analysis was performed using GraphPad Prism 3.0 software (25). All these data were expressed as mean ± SD. Differences between test samples and their respective controls were evaluated by unpaired Student’s *t*-test and considered to be significant if the *p*-Value was less than 0.05. Each experiment was performed at least thrice to validate the findings.

## Results

### Tissue-Specific Expression Analysis of AsHPX15 Gene

**Figure 1:**
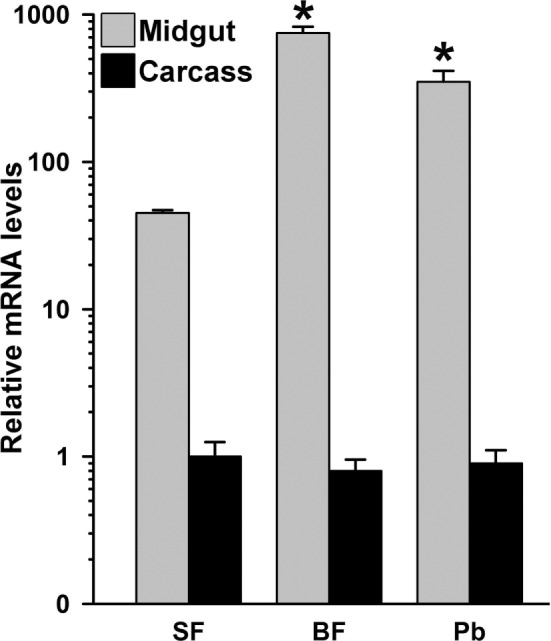
**Mosquito body compartment-specific expression of AsHPX15 gene**. Relative mRNA levels of AsHPX15 gene were analyzed in midgut and carcass of sugar fed, 24 h post normal blood fed (BF) or *Plasmodium berghei*-infected BF females. The data are presented in log_10_ scale, and significant differences in the relative mRNA levels of AsHPX15 gene are indicated by an asterisk.

To understand the organ-specific expression of AsHPX15 gene, we compared its relative mRNA levels in different body compartments. For that, we collected 24 h post uninfected or *P. berghei*-infected BF midguts and carcasses separately from 20 mosquitoes and analyzed the mRNA levels of AsHPX15 gene. We selected the 24 h post fed samples because it corresponds to the time when ookinetes invade the midgut epithelium (1, 6). Results shown in Figure 1 revealed that in SF midguts the basal levels of AsHPX15 mRNA were ~40-fold higher than carcasses. Furthermore, its expression in 24 h BF midguts was induced ~17-folds against SF midguts (Figure 1). However, the expression of AsHPX15 gene was downregulated approximately twofold in *P. berghei*-infected midguts when compared to the uninfected BF controls (*p* = 0.008, Figure 1). Interestingly, the relative mRNA levels of AsHPX15 gene were indifferent in the carcass of SF or uninfected BF mosquitoes and also remained unaffected after *Plasmodium* infection (Figure 1). From these results, we concluded that AsHPX15 is a blood induced midgut-specific gene and suppressed during the *Plasmodium* ookinete invasion of the midgut epithelium. These findings are in agreement with the previous reports where *A. gambiae* AgHPX15 gene, an ortholog of AsHPX15, is induced in BF midguts and negatively regulated after malaria parasite infection (6, 26).

**Figure 2:**
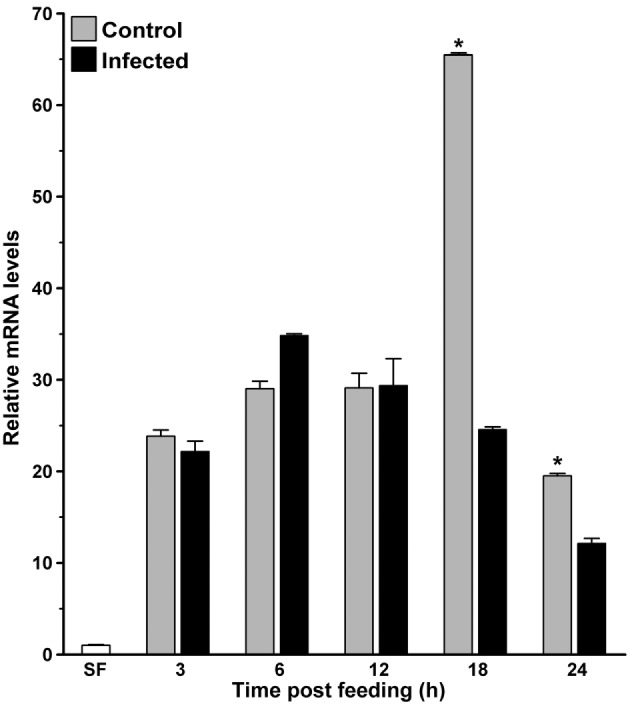
**Kinetics of AsHPX15 expression in mosquito midguts**. Relative mRNA expression levels of AsHPX15 were analyzed at different time points after normal (Control) or *P. berghei*-infected blood feeding in *Anopheles stephensi* midguts. Relative fold inductions of the gene were calculated against sugar fed midguts. Significant differences between controls and infected midguts are indicated by an asterisk.

In parallel, we also analyzed the kinetics of AsHPX15 expression in *P. berghei*-infected midguts to understand its regulation during various stages of malaria parasite development. For that, the control or *P. berghei*-infected BF midguts were collected at different time points (3, 6, 12, 18, and 24 h) after the feeding and expression levels of AsHPX15 gene were analyzed through qPCR. Results presented in Figure 2 revealed that the relative mRNA levels of AsHPX15 gene were similar in control and infected midguts for first 12 h (*p* = 0.328, *p* = 0.091, and *p* = 0.945 at 3, 6, and 12 h post blood feeding, respectively). However, there was a significant reduction of AsHPX15 mRNA levels in *P. berghei*-infected midguts at 18 h (*p* < 0.0001) and 24 h (*p* = 0.0054) post feeding when compared to the BF controls (Figure 2). These results indicated that the expression of AsHPX15 gene remains unaffected during the initial hours (up to 12 h) in infected midguts when the pre-ookinete stages of *Plasmodium* development predominate in the blood bolus (1). However, at later time points, the expression of this gene is suppressed when the mature ookinetes start invading the midgut epithelium (Figure 2).

### AsHPX15 Silencing Has a Negative Effect on *Plasmodium* Development

**Figure 3:**
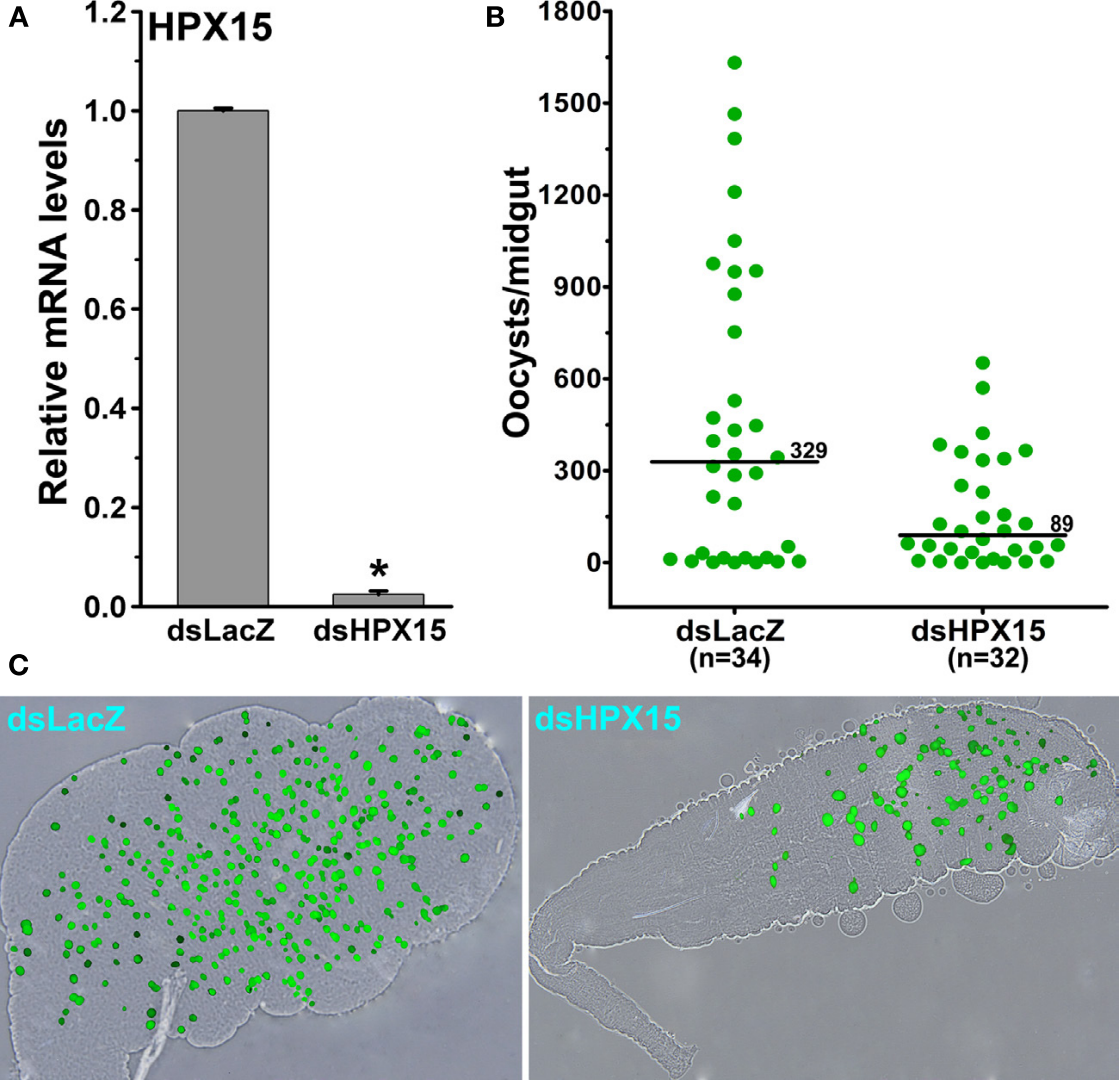
**Effect of AsHPX15 silencing on *Plasmodium berghei* development in *Anopheles stephensi***. **(A)** Relative abundance of AsHPX15 mRNA in control (dsLacZ) and silenced (dsAsHPX15) midguts that were collected 24 h post *P. berghei* infection. **(B)** Effect of AsHPX15 silencing on the number of live oocysts (green dots) in 7 days post infected blood fed midguts. Dots represent the number of parasites present in individual midguts, and the median number of oocysts is indicated by the horizontal line. Distributions are compared using the Kolmogorov–Smirnov test (*p* = 0.027); *n* = number of mosquitoes. **(C)** Representative *A. stephensi* midgut showing *P. berghei* oocysts in dsLacZ or dsHPX15-injected mosquitoes.

Our previous findings revealed that *A. gambiae* heme peroxidase AgHPX15 catalyzes the cross-linking of a mucin layer at the luminal side of the midgut epithelium (6). This cross-linked mucin barrier does not allow the bolus antigens, especially, the naturally acquired microbes, to interact with the immune reactive midgut epithelium that subsequently suppresses the activation of innate immunity in this body compartment (6, 7). Thus, we hypothesized that the reduction of AsHPX15 mRNA levels through gene-silencing approaches might accelerate the recognition and killing of *Plasmodium* by the mosquito innate immunity. To test these assumptions, we injected a group of mosquitoes with dsLacZ (controls) or dsAsHPX15 (silenced) RNA and, after 5 days, these mosquitoes were fed on *P. berghei*-infected mice. After 24 h post infected blood feeding, our analysis revealed 98% reduction of AsHPX15 mRNA levels in silenced midguts when compared to the sham-treated controls (Figure 3A).

Subsequently, we counted the number of developing oocysts in the above-mentioned control and silenced midguts after 7 days post blood feeding. Results presented in Figure 3B revealed that the variable number of oocysts were observed in controls (oocysts range 0–1,800) and silenced (oocysts range 0–600) midguts. However, the median value for the oocysts numbers in controls and AsHPX15 silenced midguts was 329 and 89, respectively. This indicated that the numbers of developing oocysts were reduced significantly in the silenced midguts against controls (Figure 3B, *p* = 0.027). Based on these data, we concluded that AsHPX15 is a natural agonist that positively regulates *Plasmodium* development inside the mosquito midgut. These findings are in agreement with the previous reports where silencing of the AgHPX15 gene in *A. gambiae* also reduced *Plasmodium* survival (6).

### AsHPX15-Silenced Midguts Induced Antiplasmodial Immunity

**Figure 4:**
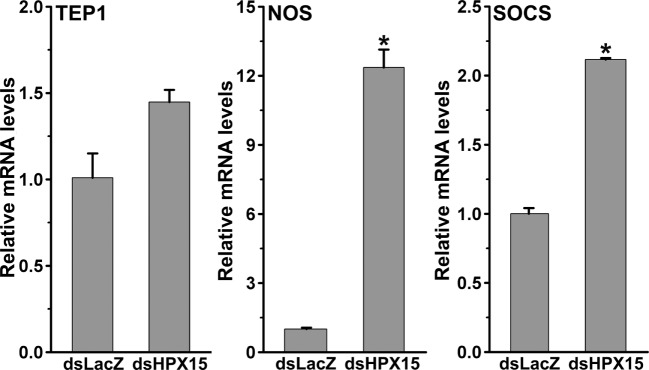
**Expression of immune genes in *Plasmodium berghei*-infected AsHPX15-silenced *Anopheles stephensi* midguts**. Relative mRNA levels of various immune genes such as, thioester-containing protein 1, nitric oxide synthase, and suppressor of cytokine signaling in 24 h post *P. berghei* infected blood fed *A. stephensi* mosquito midguts that were injected with dsLacZ or dsAsHPX15. Significant differences in mRNA levels between dsLacZ and dsAsHPX15 are indicated by asterisk.

**Figure 5:**
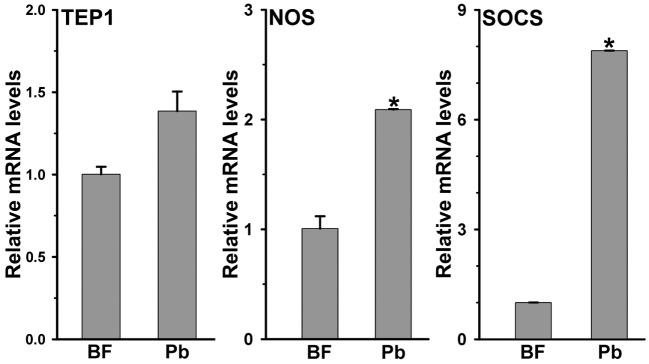
**Expression of immune genes in *Plasmodium berghei*-infected *Anopheles stephensi* midguts**. Relative mRNA expression levels of various immune genes such as, thioester-containing protein 1 (TEP1), nitric oxide synthase (NOS), and suppressor of cytokine signaling (SOCS) in 24 h post normal blood fed and *P. berghei*-infected midguts. Significant differences are indicated by asterisk.

The development of *Plasmodium* was suppressed in AsHPX15-silenced midguts (Figure 3). We assumed that the induction of an antiplasmodial immunity in the silenced midgut might be responsible for the negative regulation of *Plasmodium* development. This may be simply due to the reduced or defective cross-linking of AsHPX15-mediated mucin barrier that, in turn, allowed the interaction and recognition of parasites by the immune reactive midgut epithelium. These assumptions were confirmed through expression analysis of various known antiplasmodial genes (6, 27) in the silenced and *P. berghei*-infected BF midguts that are mentioned in Figure 3A. Our analyses revealed that the relative mRNA levels of thioester-containing protein 1 (TEP1), an antiplasmodial immune gene (27), were similar in controls and dsHPX15-injected (silenced) mosquito midguts (Figure 4, *p* = 0.106). In addition, the relative levels of TEP1 mRNA were also indifferent between uninfected or *P. berghei*-infected BF midguts (Figure 5, *p* = 0.091).

Furthermore, the comparative analysis of another antiplasmodial immune gene nitric oxide synthase (NOS) in above samples revealed that this gene was induced ~13-folds in the silenced midguts against the controls (Figure 4, *p* = 0.005). These findings suggested that the induced NOS is most probably playing an antiplasmodial role in HPX15 silenced midguts as reported before (6, 28, 29). NOS catalyzed the formation of nitric oxide, a highly diffusable and reactive immune molecule that modifies and inactivates the biological marcomolecules (23). In mosquitoes, the induction of NOS gene is regulated by the Janus kinase/signal transducers and activators of transcription (JAK*/*STAT) pathway. Previous studies revealed that the suppressor of cytokinin signaling (SOCS) is also induced in parallel to the NOS and regulates the over activation of JAK/STAT pathway (6, 15, 28, 29). Thus, we also analyzed the induction of SOCS gene in the above-mentioned AsHPX15 silenced midguts to understand the activation of NOS through JAK/STAT pathway. Results presented in Figure 4 revealed that the SOCS gene was induced approximately twofold in the silenced midguts against controls (*p* = 0.001). Thus, the induction of NOS, an effector gene, and SOCS, a suppressor gene, indicated that the activation of JAK/STAT pathway might regulate antiplasmodial immunity in AsHPX15 silenced midguts. In addition, both NOS and SOCS genes were also induced twofold and eightfold, respectively in *Plasmodium*-infected unsilenced midguts when compared to the BF controls (Figure 5, *p* = 0.011 for NOS and *p* < 0.0001 for SOCS). These results collectively indicated that, in general, the JAK/STAT pathway is induced in *Plasmodium*-infected midguts (Figure 5). However, an additional induction of the same pathway in HPX15-silenced midguts seems to be the major regulator of *Plasmodium* development (Figure 4).

In conclusion, the absence of HPX15-catalyzed mucin barrier in infected midgut provides a better opportunity for the innate immune system to recognize the malaria parasite that, in turn, activates JAK/STAT pathway to regulate *Plasmodium* development negatively.

## Discussion

Mosquito midgut is the organ for digestion and also plays an important role in immunity. The midgut is housed by a large variety of microbes as well as it is the first organ that encounters blood-borne pathogens. The mutualistic association between the midgut and naturally acquired microbes is important for digestion and nutrition (6, 7, 30). Endogenous microbes proliferate in the lumen of BF midgut. Therefore, this organ is equipped with the mechanism(s) that maintain(s) a remarkable balance between microbial homeostasis and immunity against the bolus antigens. One of the mechanisms that balances these events has been discussed in the African malaria vector *A. gambiae* (6, 7). In this mosquito, a tyrosine cross-linked mucins barrier is formed on the luminal surface of the BF midgut that blocks the recognition of bolus antigens or microbial elicitors by the immune reactive epithelial cells. Importantly, the cross-linking of mucins barrier is catalyzed by a heme peroxidase AgHPX15, which is also a blood feeding-induced midgut-specific gene [Ref. (6, 7); also Figure 1 of this study].

Gene silencing studies carried in *A. gambiae* revealed that midgut bacteria as well as *Plasmodium* is recognized and killed by the midgut immunity in AgHPX15-silenced midguts. This effect was due to the reduced cross-linking of the mucin barrier, recognition of lumen bacteria and *Plasmodium* by the immune reactive midgut epithelium, and induction of antigen-specific innate immunity. It is of note that the suppressive effects of AgHPX15 silencing were identical against *P. falciparum* (human malaria) and *P. berghei* (murine malaria) (6).

Recent studies from our laboratory identified AgHPX15 ortholog in the Indian malaria vector *A. stephensi* and named it as AsHPX15 (10, 11). Interestingly, AsHPX15 and AgHPX15 are true orthologs and exhibit identical characteristics in several ways. For example, both orthologs are (a) midgut-specific and induced after blood feeding, (b) unique anopheline lineage-specific genes and do not have their orthologs in arthropods, including other mosquitoes, and human, (c) highly conserved and their putative ortholgs are present in the genome of 17 other worldwide distributed anophelines and share 65–99% amino acids identity, and (d) having same putative transcription factors binding sites in their regulatory region (10, 11). We assumed that due to the shared common features, *A. stephensi* AsHPX15 and *A. gambiae* AgHPX15 should be functionally identical in terms of modulating midgut immunity against bolus antigens. If it is true, then HPX15 can be considered as a common target to manipulate the midgut antiplasmodial immunity of anopheline mosquitoes (10, 11).

Thus, in the present study, we explored the role of *A. stephensi* AsHPX15 gene in regulation of *Plasmodium* development and replicating the findings reported earlier in case of AgHPX15 (6). We found that AsHPX15 gene is exclusively expressed in *A. stephensi* midgut, induced as early as 3 h post blood feeding and its mRNA levels remain elevated for first 12 h post blood feeding. Interestingly, the expression of AsHPX15 is significantly reduced during ookinete invasion of the midgut epithelium (24 h post infected blood feeding) (Figures 1 and 2). These findings are in agreement with the previous reports where AgHPX15 gene followed similar expression profile in BF midguts (6).

Furthermore, our analysis of AsHPX15 role in regulation of *Plasmodium* development revealed that parasite number was drastically reduced in silenced midguts (Figure 3). We believed that the absence of HPX15 protein would have resulted in the formation of a defective mucin barrier and that, in turn, induced antiplasmodial immunity in silenced midguts. Interestingly, we found that NOS, a downstream effector gene of JAK/STAT pathway, is highly induced in these samples (Figure 4) and might be one of the important negative regulators of *Plasmodium* development in the similar way as reported in *A. gambiae* (6). These results collectively indicated that the silencing of HPX15 gene has negative effects on *Plasmodium* development at least in two anophelines, *A. stephensi* and *A. gambiae*.

As we discussed earlier, the formation of HPX15-mediated mucin barrier is important for suppressing the midgut immunity against bolus antigens. It is believed that this mechanism is necessary to augment the process of digestion without activating immunity against food particles. Thus, the low-immunity zone created in this body compartment is also exploited by the pathogens to support their own development. Our study demonstrated that the HPX15 gene is conserved in anopheline mosquitoes in terms of its functional properties to modulate antiplasmodial immunity of the midgut. Therefore, it upholds a promising future to block mosquito cycle of *Plasmodium* development.

## Conclusion

HPX15 is a unique, highly conserved protein among 19 anophelines. Its functional properties in terms of regulating the malaria parasite development are identical in two anophelines, *A. stephensi* and *A. gambiae*. Thus, we propose that anopheline HPX15 might serve as a “potent candidate” that can be targeted to manipulate mosquito vectorial capacity and blocking the transmission of malaria infection.

## Ethics Statement

The protocol for using mice in this study was approved by Institutional Animal Ethics Committee (protocol number: IAEC/RES/13/01/REV/15/1) under the guidelines of Committee for the Purpose of Control and Supervision of Experiments on Animals, Minisrty of Environment, Forests and Climate Change, Government of India.

## Author Contributions

MK, LG, and SK designed experiments for this work. MK, TC, PK, KG, and RD collected samples to perform experiments. MK, TC, PK, KG, RD, LG, and SK performed expression kinetics of different immune genes. MK, TC, PK, LG, and SK performed HPX15-silencing experiments. MK, LG, VK, and SK analyzed these data and wrote the manuscript with input from all authors. All the authors read and approved the manuscript.

## Conflict of Interest Statement

The authors declare that the research was conducted in the absence of any commercial or financial relationships that could be construed as a potential conflict of interest. The reviewer AT and handling editor declared their shared affiliation, and the handling editor states that the process nevertheless met the standards of a fair and objective review.
